# Identification of Large Adenovirus Infection Outbreak at University by Multipathogen Testing, South Carolina, USA, 2022

**DOI:** 10.3201/eid3002.230623

**Published:** 2024-02

**Authors:** Marco E. Tori, Judith Chontos-Komorowski, Jason Stacy, Daryl M. Lamson, Kirsten St. George, Avril T. Lail, Heather A. Stewart-Grant, Linda J. Bell, Hannah L. Kirking, Christopher H. Hsu

**Affiliations:** South Carolina Department of Health and Environmental Control, Columbia, South Carolina, USA (M.E. Tori, L.J. Bell);; Centers for Disease Control and Prevention, Atlanta, Georgia, USA (M.E. Tori, H.L. Kirking, C.H. Hsu);; University of South Carolina Student Health Services, Columbia (J. Chontos-Komorowski, J. Stacy, A.T. Lail, H.A. Stewart-Grant);; New York State Department of Health, Albany, NY, USA (D.M. Lamson, K. St. George)

**Keywords:** adenoviruses, outbreaks, students, universities, public health, COVID-19, respiratory infections, severe acute respiratory syndrome coronavirus 2, SARS-CoV-2, SARS, coronavirus disease, zoonoses, viruses, coronavirus, South Carolina, United States

## Abstract

Using multipathogen PCR testing, we identified 195 students with adenovirus type 4 infections on a university campus in South Carolina, USA, during January–May 2022. We co-detected other respiratory viruses in 43 (22%) students. Continued surveillance of circulating viruses is needed to prevent virus infection outbreaks in congregate communities.

Human adenovirus (HAdV) infections can cause a range of symptoms but most commonly result in respiratory illnesses (*1*). Most HAdV infections are not clinically severe; however, more serious illness can occur (*2*,*3*). A total of 51 recognized HAdV serotypes and >100 genotypes (classified into 7 species, HAdV-A–G) have been characterized globally (*4*). Because testing does not change clinical management, persons with HAdV infections often do not receive a virus infection diagnosis. If adenovirus testing is available, it is usually performed as part of a multipathogen PCR panel. Adenovirus infection outbreaks caused by transmission through respiratory droplets and fomites have been reported in various congregate settings, including nursing homes (*5*), military recruit barracks (*6*–*8*), and college campuses (*9*–*11*). The incubation period varies from 2–14 days.

In early February 2022, a university campus in South Carolina, USA, notified its regional health department of 4 students with HAdV infections who had sought care for respiratory symptoms the previous week at student health services (SHS). Nasopharyngeal swab specimens were collected and tested by using a multipathogen PCR panel for respiratory pathogens; HAdV was detected in all 4 patient samples. SHS contacted the South Carolina Department of Health and Environmental Control and the Centers for Disease Control and Prevention (CDC) to request typing of the HadV specimens to determine if >1 HadV type was circulating. Partial genomic sequencing showed that HadV was the same type in all 4 specimens. Cases of HadV infections continued to increase on campus; therefore, the university, state and local health departments, and CDC investigated the scope of the outbreak. The timing of this outbreak during the COVID-19 pandemic enabled unique observations and responses. We describe the outbreak, the university’s response intended to prevent further transmission, and implications of HadV infection outbreaks in congregate settings, such as universities. The South Carolina Department of Health and Environmental Control Institutional Review Board deemed this work was non–human subjects research. This activity was reviewed by CDC and was conducted consistent with applicable federal law and CDC policy.

## The Study

We analyzed laboratory and exposure data from symptomatic students who sought care at the university’s health clinic during January 1–May 31, 2022. SHS staff collected nasal or nasopharyngeal swab samples from students and tested those specimens by using BioFire RSP 2.1 multiplex PCR (bioMérieux, https://www.biomerieux.com). We defined positive cases as students who manifested respiratory or constitutional symptoms and were HadV-positive in the multipathogen PCR panel. 

SHS routinely collected demographic information, symptoms, and medical history from students seeking care at the university clinic. Those data were supplemented from March 22–May 10, 2022, by using focused call-back interviews of students who had confirmed HadV infections to identify potential transmission events and locations. We used the aggregate interview data to examine student behaviors and activities that might have been associated with transmission events.

We considered students who tested positive for HadV plus another respiratory pathogen on the multipathogen panel to have pathogen co-detections. We compared demographic characteristics, symptoms, and illness severity between students who had co-detections and those who were only infected with HadV by using *t*-tests.

During January 1–May 31, 2022, a total of 687 students were tested by using the respiratory multipathogen PCR panel after seeking care at SHS for acute respiratory or systemic symptoms. Of those 687 students, 195 (28.4%) tested positive for HadV; HadV infections were distributed evenly between men and women. The median age of infected students was 19 years (range 18–24 years) (Table 1). The most common symptoms reported by students were sore throat (85%), cough (76%), fever (75%), and headache (62%). Nausea and vomiting were reported by 27% of students; conjunctivitis was reported by 10% of students. No known emergency department visits, hospitalizations, or deaths were reported among any HadV-infected students. HadV-4 was identified in 30 swab specimens by partial genomic sequencing of the hexon gene (Appendix). We randomly selected 8 of those 30 sequences for whole-genome sequencing and performed phylogenetic analyses (Appendix Figure). 

**Table 1:** Characteristics of university students in study identifying a large adenovirus infection outbreak by multipathogen testing, South Carolina, USA, 2022*

Characteristics	Adenovirus infections
Total no. infected students	195
Sex	
M	97 (50)
F	98 (50)
Median age, y (range)	19 (18–24)
Academic class	
Freshman	76 (39)
Sophomore	58 (30)
Junior	33 (17)
Senior	25 (13)
Graduate student	3 (1)
Residence	
On-campus dormitory	115 (59)
On-campus apartment	6 (3)
Off campus	68 (35)
Unknown	6 (3)
Area of academic study	
Prebusiness or business	41 (21)
Finance	17 (9)
Biology	17 (9)
Psychology	13 (7)
Public health	9 (5)
Sports/entertainment management	8 (4)
Undeclared	8 (4)
Political science	7 (4)
All others	75 (38)
Symptoms†	
Cough	149 (76)
Sore throat	166 (85)
Fever	146 (75)
Headache	120 (62)
GI	52 (27)
Conjunctivitis	20 (10)
Smoking or vaping	30 (15)
Comorbidities	
Asthma	14 (7)
Immunocompromised‡	2 (1)
Other or not reported	179 (92)
Co-detected respiratory viruses§	
Human rhinovirus/enterovirus	28 (14)
Seasonal coronavirus¶	9 (5)
SARS-CoV-2	8 (4)
Parainfluenza, types 2–4	6 (3)
Influenza A	1 (1)
RSV	1 (1)
Human metapneumovirus	1 (1)
Adenovirus typing by hexon gene sequencing	
Human adenovirus type 4	30 (15)
Not typed#	165 (85)

*Values are no. (%) except as indicated. †Students could report multiple symptoms. ‡Immunocompromised because of methotrexate or immunomodulatory agent treatment. §Other respiratory viruses were detected by using multipathogen PCR. A total of 43 students tested positive for human adenovirus (HAdV) and >1 other respiratory pathogen. Ten of 43 students tested positive for HAdV and >2 other pathogens; 5 of those students had HAdV, SARS-CoV-2, and human rhinovirus/enterovirus. One student tested positive for HAdV, SARS-COV-2, parainfluenza 2, and human rhinovirus/enterovirus. Additional testing, including typing or genomic sequencing, was not performed on specimens that were negative for HAdV. ¶Seasonal coronavirus types were OC43, 229E, NL63, and HKU1. #Typing not attempted or not possible.

Weekly case counts increased slowly during January–February and more rapidly after the week of March 6 (university spring break) (Figure). Rapid and detailed attention to surface decontamination in academic and residential buildings on campus was reported in February and early March. Mandatory masking recommendations as part of COVID-19 mitigation efforts were lifted in March. Cases fell precipitously after students left campus for summer break (week of May 1); no cases were reported after May 10, 2022. The outbreak investigation was closed on June 7 after 2 full incubation periods (total of 28 days).

**Figure:**
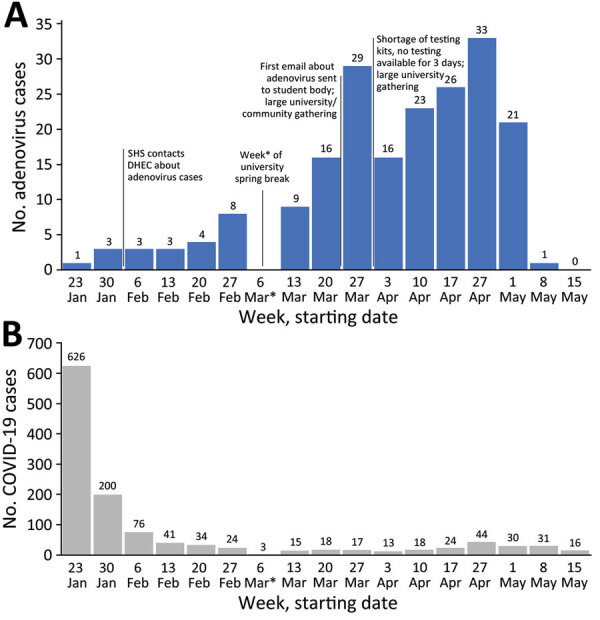
Number of adenovirus (A) and COVID-19 (B) cases at a university campus in South Carolina in study identifying a large adenovirus infection outbreak by multipathogen testing, South Carolina, USA, January 1–May 31, 2022. Numbers above bars indicate number of cases at each weekly time point. Vertical lines between bars indicate timelines of university events affecting the outbreak. Asterisks indicate the week of university spring break, after which weekly case counts began to rapidly increase. DHEC, Department of Health and Environmental Control; SHS, student health services.

Of all HadV-infected students, infections occurred most in first-year (39%) and second-year (30%) students. Most (115 [59%]) students lived in on-campus dormitories. Three of 22 affected dormitories had >10 students with confirmed HadV infections during the outbreak. We did not observe a preponderance of one academic area of study that might suggest clustering according to academic departments.

Among the 195 students who tested positive for HadV, >1 other respiratory pathogen was also detected in 43 (22%) students (Table 2). The most common co-detected viruses were human rhinovirus/enterovirus (28 [65%]), seasonal coronaviruses (9 [21%]), and SARS-CoV-2 (8 [19%]). Most (42 [98%]) HadV-infected students who had co-detected viruses reported a sore throat, compared with 124 (82%) students infected with only HadV (p = 0.001). More HadV-only–infected students (13%) reported conjunctivitis than students who had co-detected viruses (2%; p = 0.001). We did not observe a substantial difference in disease severity (i.e., need for intravenous fluids) among HadV-only–infected students compared with those who had virus co-detections (4% vs. 2%; p = 0.07).

**Table 2:** Comparison of university students with HAdV infection only and those with respiratory virus co-detections in study identifying a large adenovirus infection outbreak by multipathogen testing, South Carolina, USA, 2022*

Characteristics	HAdV infection only	HAdV + co-detected respiratory virus†	p value
No. students	152	43	NA
Sex			
F	70 (46)	28 (65)	0.002
M	82 (54)	15 (35)	0.03
Median age, y (range)	19 (18–24)	19 (18–22)	NA
Residence			
On campus dormitory	85 (56)	30 (66)	0.09
Off campus	59 (39)	11 (25)	0.09
Symptoms			
Cough	113 (74)	36 (84)	0.16
Sore throat	124 (82)	42 (98)	0.001
Fever	116 (76)	30 (70)	0.41
Headache	90 (59)	30 (70)	0.19
Nausea or vomiting	44 (29)	8 (19)	0.14
Conjunctivitis	19 (13)	1 (2)	0.001
Severe infection	6 (4)	1 (2)	0.07
Smoking or vaping	15 (11)	5 (12)	0.75

*Values are no. (%) except as indicated. p values were obtained from 2-sided *t*-tests. HAdV, human adenovirus; NA, not applicable. †One student was HAdV positive in March, then had recurrent symptoms in early May and was found to be both HAdV and parainfluenza 3 positive. The student was included in the HAdV infection only column and not in the co-detection column. This student’s clinical course and timeline were more consistent with a new parainfluenza infection; the detection of adenovirus was likely attributable to prolonged virus DNA shedding, not a new infection. This group does not include students with Epstein-Barr virus or group A *Streptococcus* infections.

The university clinic staff began interviewing students with confirmed HadV infection on March 21. Interviews were completed for 96 of 121 students with HadV (79% response rate) before the end of the outbreak. Most (62 [65%]) students did not know where they had acquired infection; 9 (9%) students had a known exposure to someone with confirmed HadV infection, and 18 (19%) students had exposure to someone with similar symptoms before illness onset. Seventy-five (78%) students had not traveled away from campus before their symptoms began. We were unable to link infections to campus or off-campus locations because of limited sample size.

## Conclusions

This outbreak of respiratory illness attributed to HadV-4 was among the largest described HadV outbreaks on a university campus (*9*,*12*); symptoms and transmission were similar to other large HadV outbreaks in congregate settings (*13*). Infected students were mostly freshmen and sophomores living in dormitories, highlighting increased transmission in close university settings. The outbreak occurred during the COVID-19 pandemic, and detection of HadV might have been delayed without availability of multipathogen testing. The outbreak on campus appeared to end when student density decreased during summer break, and further transmission was not observed among the limited number of students remaining on campus.

SHS was able to detect and respond quickly to a potentially serious virus infection outbreak by using multipathogen testing. Furthermore, this testing enabled identification of students who were infected with multiple pathogens. In addition to SARS-CoV-2, many other respiratory viruses can be detected in university students, including those that cause illness and outbreaks. As universities move beyond COVID-19 as the main public health priority affecting students and campuses, renewed attention to other pathogens is needed (*14*). Continued surveillance of circulating viruses in congregate communities remains critical for ongoing risk communication and prevention efforts.

Appendix Additional information for identification of large adenovirus infection outbreak at university by multipathogen testing, South Carolina, USA, 2022.
