# Supplementary material for: Identification of Large Adenovirus Infection Outbreak at University by Multipathogen Testing, South Carolina, USA, 2022
Source: Emerg Infect Dis. 2024 Feb;30(2):358–62. doi: 10.3201/eid3002.230623 (PMC10826757; doi:10.3201/eid3002.230623)
Supplement: Appendix — Additional information for identification of large adenovirus infection outbreak at university by multipathogen testing, South Carolina, USA, 2022. [file 23-0623-Techapp-s1.pdf]

Article DOI: https://doi.org/10.3201/eid3002.230623

EID cannot ensure accessibility for supplementary materials supplied by authors. Readers who have difficulty accessing supplementary content should contact the authors for assistance.

Identification of Large Adenovirus Infection Outbreak at University by Multipathogen Testing, South Carolina, USA, 2022

Appendix

Additional Methods

Genetic Typing and Whole Genome Sequencing

At 3 different time points during the investigation, a subsample of specimens that tested positive for human adenovirus (HAdV) were randomly selected for genetic typing at the New York State Department of Health Wadsworth Center. Specifically, HAdVs were typed by performing bidirectional sequencing of hypervariable regions 1–6 of the hexon gene (*1*); types were identified by using BLAST analysis (https://blast.ncbi.nlm.nih.gov), and sequences were deposited in GenBank. A random subsample of typed specimens also underwent whole genome sequencing at the Wadsworth Center (*2*,*3*).

Results

Thirty student swab specimens that were sent for typing were identified as HAdV-4. One typed specimen had a single nucleotide polymorphism that caused an amino acid change in the hexon gene sequence. Among the 30 typed specimens, 8 sequences were randomly selected for whole genome sequencing. Phylogenetic analysis was performed for the 8 sequences from this study and reference sequences downloaded from GenBank (Appendix Figure). Those 8 sequences are available in GenBank (accession nos. OP785759–66).

References

1. Okada M, Ogawa T, Kubonoya H, Yoshizumi H, Shinozaki K. Detection and sequence-based typing of human adenoviruses using sensitive universal primer sets for the hexon gene. Arch Virol. 2007;152:1–9. PubMed https://doi.org/10.1007/s00705-006-0842-8

2. Kajon AE, Erdman DD. Assessment of genetic variability among subspecies b1 human adenoviruses for molecular epidemiology studies. Methods Mol Med. 2007;131:335–55. PubMed https://doi.org/10.1007/978-1-59745-277-9_23

3. Lamson DM, Kajon A, Shudt M, Girouard G, St George K. Detection and genetic characterization of adenovirus type 14 strain in students with influenza-like illness, New York, USA, 2014–2015. Emerg Infect Dis. 2017;23:1194–7. PubMed https://doi.org/10.3201/eid2307.161730

4. Kajon AE, Lamson DM, Bair CR, Lu X, Landry ML, Menegus M, et al. Adenovirus type 4 respiratory infections among civilian adults, northeastern United States, 2011–2015. Emerg Infect Dis. 2018;24:201–9. PubMed https://doi.org/10.3201/eid2402.171407

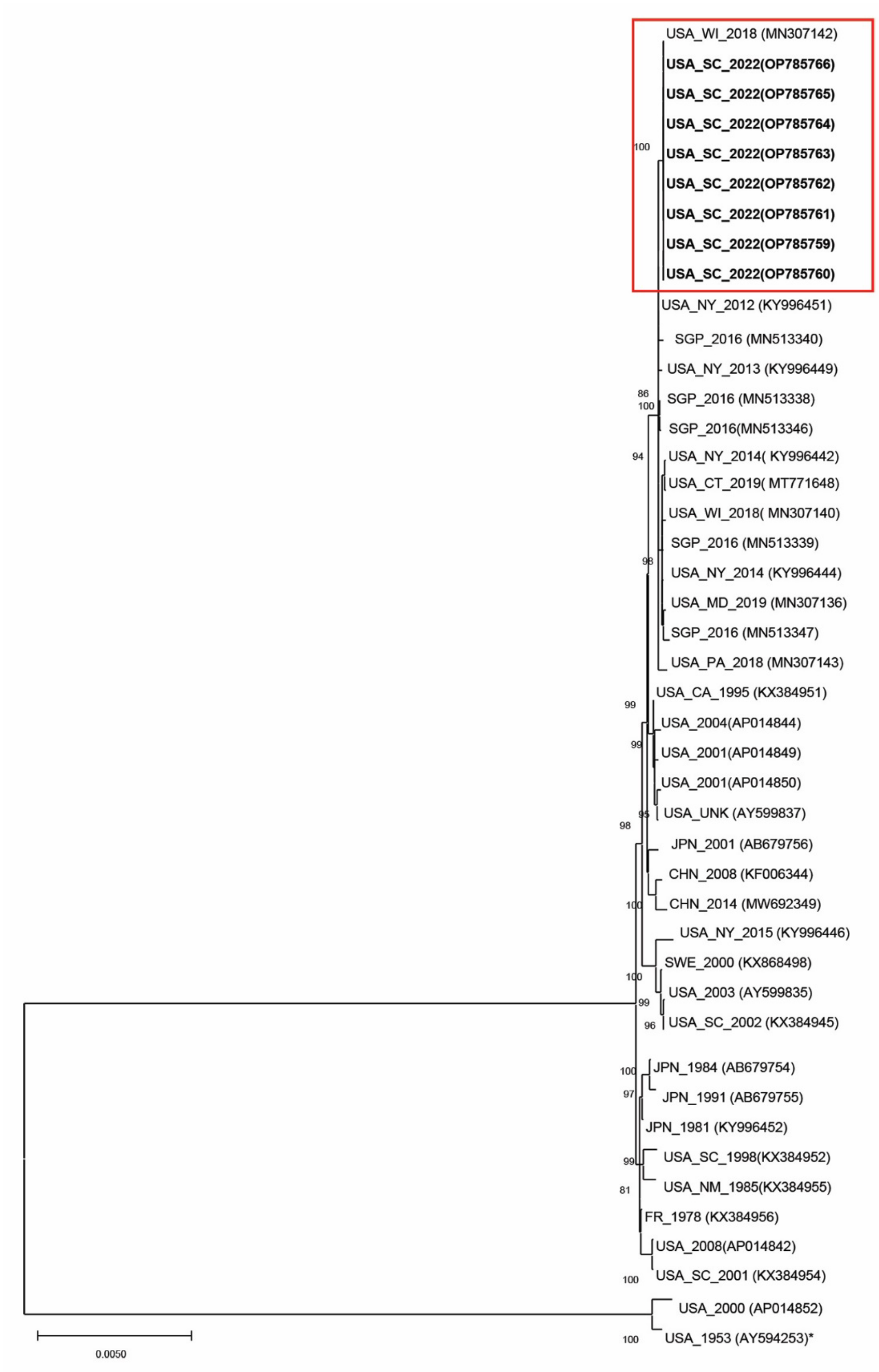

Appendix Figure. Phylogenetic analysis of complete genome sequences from a human adenovirus (HAdV) infection outbreak in South Carolina, USA, compared with HAdV-4 sequences from GenBank. Red box indicates isolates from the outbreak described in this study (labeled SC) that were identical to a sequence from Wisconsin (WI), USA. Samples were processed for whole genome sequencing and

sequenced on the Illumina MiSeq instrument (*4*). Assembled genomes were aligned to other sequences of the same type obtained from GenBank (*4*). Phylogenetic tree was constructed by using the maximum-likelihood method and Kimura 2-parameter model to display associations among human adenovirus isolates from this study (GenBank accession nos. OP785759–66) and select reference sequences downloaded from GenBank. Tree was created in IQ-TREE (http://www.iqtree.org) by using the general time reversible model plus empirical base frequencies (proportion of invariable sites plus gamma distribution 4 plus nonparametric bootstrap with 100 replicates) and displayed in MEGA X (https://www.megasoftware.net). Accession numbers for reference strains are provided in parentheses next to the country, strain designation, and year of isolation, if known. Nodes with bootstrap values >70 are shown. Asterisk indicates prototype strain RI-67. Scale bar indicates nucleotide substitutions per site.
